# The Value of a Time-Table

**Published:** 1917-03-24

**Authors:** 


### THE VALUE OF A TIME-TABLE.

When one comes to think of it, almost the whole utility and value of a hospital depend upon the strict observance of the recognised official time-table. The hours and times checked at the entrance lodge, or in the kitchen, are in their way just as important as those holding good in the wards and in the operating theatres. It is not too much to say that the present great difficulties in shipping circles are the outcome of a sadly-disturbed time-table: our ships, and the ships of other nations, have had to ignore their time-tables, with what result we all know. And in hospital life how very true it is that the value of a time-table cannot be over-estimated. If the cook is late with the "light diets," the patients and the nurses have an uncomfortable afternoon. Of course, the cook will blame the fishmonger or the poultryman for being late—for treating the time-table with contempt. Again, it is the physician who knows, above all others, what is the dire seriousness should certain developments and conditions for which he has striven not occur at a certain hour; if he is to pull his difficult case through, something must happen by such-and-such a time. The out-patient department would be as excited and chaotic as a country fair were it not for a steady and true application of the time-table rules. Of all centres in the world the value of a time-table is not more palpable than in a hospital.

